# Genetic diversity analysis in wheat cultivars using SCoT and ISSR markers, chloroplast DNA barcoding and grain SEM

**DOI:** 10.1186/s12870-023-04196-w

**Published:** 2023-04-11

**Authors:** Heba H. Abouseada, Al-Safa H. Mohamed, Samir S. Teleb, Abdelfattah Badr, Mohamed E. Tantawy, Shafik D. Ibrahim, Faten Y. Ellmouni, Mohamed Ibrahim

**Affiliations:** 1 Department of Botany, Faculty of Science, Ain Shams University, Cairo, Egypt; 2 Botany and Microbiology Department, Faculty of Science, Zagazig University, Zagazig, 44519 Sharqia Egypt; 3 Botany and Microbiology Department, Faculty of Science, Helwan University, Cairo, Egypt; 4 Agricultural Genetic Engineering Research Institute (AGERI), Agricultural Research Center (ARC), Giza, Egypt; 5 Botany Department, Faculty of Science, Fayoum University, 63514 Fayoum, Egypt

**Keywords:** Wheat (*Triticum aestivum* L.), Genetic diversity, Molecular markers, Chloroplast DNA barcodes, Grain exomorphic characteristics

## Abstract

**Background:**

Wheat is a major cereal that can narrow the gap between the increasing human population and food production. In this connection, assessing genetic diversity and conserving wheat genetic resources for future exploitation is very important for breeding new cultivars that may withstand the expected climate change. The current study evaluates the genetic diversity in selected wheat cultivars using ISSR and SCoT markers, the *rbc*L and *mat*K chloroplast DNA barcoding, and grain surface sculpture characteristics. We anticipate that these objectives may prioritize using the selected cultivars to improve wheat production. The selected collection of cultivars may lead to the identification of cultivars adapted to a broad spectrum of climatic environments.

**Results:**

Multivariate clustering analyses of the ISSR and SCoT DNA fingerprinting polymorphism grouped three Egyptian cultivars with cultivar El-Nielain from Sudan, cultivar Aguilal from Morocco, and cultivar Attila from Mexico. In the other group, cultivar Cook from Australia and cultivar Chinese-166 were differentiated from four other cultivars: cultivar Cham-10 from Syria, cultivar Seri-82 from Mexico, cultivar Inqalab-91 from Pakistan, and cultivar Sonalika from India. In the PCA analysis, the Egyptian cultivars were distinct from the other studied cultivars. The *rbc*L and *mat*K sequence variation analysis indicated similarities between Egyptian cultivars and cultivar Cham-10 from Syria and cultivar Inqalab-91 from Pakistan, whereas cultivar Attila from Mexico was distinguished from all other cultivars. Combining the data of ISSR and SCoT with the *rbc*L and *mat*K results retained the close resemblance among the two Egyptian cultivars EGY1: Gemmeiza-9 and EGY3: Sakha-93, and the Moroccan cultivar Aguilal, and the Sudanese cultivar El-Nielain and between Seri-82, Inqalab-91, and Sonalika cultivars. The analysis of all data distinguished cultivar Cham-10 from Syria from all other cultivars, and the analysis of grain traits indicated a close resemblance between cv. Cham-10 from and the two Egyptian cultivars Gemmeiza-9 and Sakha-93.

**Conclusions:**

The analysis of *rbc*L and *mat*K chloroplast DNA barcoding agrees with the ISSR and the SCoT markers in supporting the close resemblance between the Egyptian cultivars, particularly Gemmeiza-9 and Sakha-93. The ISSR and SCoT data analyses significantly expressed high differentiation levels among the examined cultivars. Cultivars with closer resemblance may be recommended for breeding new wheat cultivars adapted to various climatic environments.

**Supplementary Information:**

The online version contains supplementary material available at 10.1186/s12870-023-04196-w.

## Background

Wheat (*Triticum* L.) species have been developed into thousands of polyploid cultivars that vary in the number of chromosomes from the diploid original primitive types [1]. Bread wheat (*Triticum aestivum* L.) is a hexaploid species (2n = 6x = 42) of three genomes (AABBDD), containing three related ancestral genomes, each having 14 chromosomes [2, 3]. Developed cultivars were classified based on horticultural demand, food uses, and texture. The development and growth of thousands of common wheat cultivars have been achieved worldwide. However, new and improved cultivars are always required to increase wheat grain yield and meet the food needs of the ever-expanding human population [4, 5].

Climate changes shall impact the earth's environment through temperature fluctuations, changing rainfall distribution, loss of soil fertility, increased salinity, biological stresses, increased pollution, and declining biodiversity [6]. The brutality of climate change on crop production may be maximized because more than one factor affects plant growth and development [7, 8]. Assessing and conserving wheat genetic resources for future exploitation is very important [9, 10]. Meanwhile, pre-breeding material and cultivars are exploited in genomics-assisted breeding strategies to improve the productivity of wheat cultivars [11].

DNA markers have been developed and applied to assess genetic diversity in crop plants [12]. The inter simple sequence repeats (ISSRs) developed by Bornet and Branchard [13] involved the amplification of genomic segments flanked by inversely oriented and closely spaced microsatellite sequences by a single primer or a pair of primers based on SSRs anchored 5' or 3' with 1–4 purine or pyrimidine residues. The Start Codon Targeted (SCoT) sequence,, a dominant and reproducible marker like ISSR, is based on a short conserved region in plant genes surrounding the ATG translation start codon. SCoT uses a single 18-mer primer in a polymerase chain reaction (PCR) assays and requires an annealing temperature of 50 °C [14]. The ISSR and SCoT polymorphism has been used in genetic resource differentiation, cultivar characterization, and marker-assisted breeding programs in many plants; examples include alfalfa and Egyptian clover [15], *Nigella sativa* [16], *Medicago sativa* [17], *Pistacia lentiscus* [18], *Moringa oleifera* [19], *Lathyrus* species [20], *Crepidium acuminatum* [21], maize [22], *Hordeum* [23], and smooth bromegrass [24, 25].

Etminan et al. [26] evaluated the genetic variation of a mini-core collection of breeding lines and landraces of durum wheat germplasm using 15 ISSR and 6 SCoT markers combined with studying agro-morphological traits. Pour-Aboughadareh et al. [27] also used 15 SCoT primers to assess the genetic diversity of 70 accessions of Iranian *Triticum* species. The SCoT markers were also employed by Mohamed et al. [28] to genetically characterize 14 cultivars of wheat from North Africa. Also, SCoT was used to discriminate eight wheat Asian cultivars [29]. Genetic diversity and population structure of 80 *Triticum urartu* accessions were investigated using SCoT and CBDP markers [30]. Molecular variability and relationships within the set of 91 samples of *Triticum aestivum*, *Aegilops cylindrica*, and *Aegilops crassa* species were estimated by using CBDP and SCoT markers [31]. In the same context, the genotypic and phenotypic diversity of 96 durum wheat genotypes were assessed using CBDP and ISSR markers [32].

DNA barcoding for identifying plant species was introduced by Hebert et al. [33] and was further developed, as reported by Hollingsworth et al. [34]. The chloroplast genes' large subunit of the ribulose-bisphosphate carboxylase (*rbc*L) and maturase kinase (*mat*K) are indispensable barcodes for plant species. Additionally, the chloroplast spacer, the *psb*A and the ribosomal internal transcripted spacer (ITS) sequences are commonly employed barcodes at the species level [35]. Moreover, DNA barcoding provides insights into genetic diversity and comparative investigations in studied populations [36]. Recently, DNA barcoding has become a valuable technique for assessing biodiversity in phylogenetic reconstruction and plant evolution [37–39]. Combined nuclear and chloroplast DNA sequences were used to barcode the major forage plants [15, 18, 40]. Knowledge about the genetic diversity of 12 bread wheat cultivars is limited, not only among Northern African varieties but also in cultivars in different countries. The importance of selecting the prementioned wheat cultivars lies in their capability for adaptation to the extreme climatic conditions in the countries where these cultivars were developed [28, 29]. In the same context, several pilot and preliminary measurements, including morphological characteristics (ca. grain length, width, color, etc.), physical analyses (ca. moisture %, falling number, ash content, wet gluten, dry gluten, etc.), and grain weight (ca. the weight of one and 20 grains) (data not shown) were performed on previosuly published wheat cultivars [28, 29] and revealed focusing on the 12 selected studied cultivars. Also, selected genotypes harbor abiotic resistant genes beside other genes encode transcription factors play a pivotal role in plant growth and development [28, 29].

The grains of cereals have diagnostic features that help in the separation and characterization of cultivars and their contribution to the quality and yield for consumers, farmers, and plant breeders [41–43]. A pronounced degree of similarity among the four genotypes of cereal bowls was achieved by SEM screening of the grain surface [44].

The current study aims to estimate the genetic diversity in 12 selected wheat cultivars of different origins by applications of molecular markers and classical morphological markers. Several marker types are employed; ISSR and SCoT markers, the *rbc*L and *mat*K chloroplast DNA barcoding, and grain surface sculpture characteristics were addressed in evaluating the genetic diversity. We anticipate that these objectives may prioritize using the selected cultivars to improve wheat production. Ongoing advances in cultivars' differentiation significantly accelerate the breeding of more productive cultivars to enhance wheat production to achieve the goal of doubling the yield by 2050 [11]. Using wheat cultivars of different origins may lead to successfully breeding cultivars adapted to a broad spectrum of climatic environments. Moreover, the integration and complementation of executed molecular and morphological analyses trigger a more comprehensive evaluation of the genetic variability in the studied cultivars.

## Results

### ISSR and SCoT fingerprinting

**Fig. 1:**
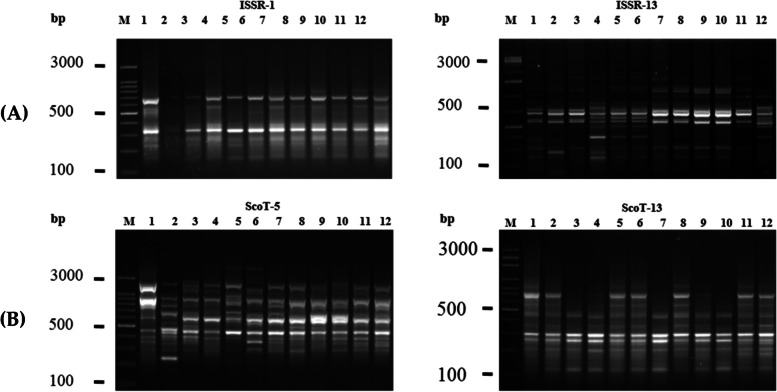
Photographs of some representative agarose gel electrophoresis of PCR amplicons of A) ISSR and B) SCoT primers. M = DNA size marker in bps (Cat. No. SB_07-11-0000S, MEDIBENA Life Science & Diagnostic Solutions, Vienna, Austria). Numbers from 1 to 12 refer to the sampling numbers of the studied cultivars. The primer codes are listed in Table 2. Fully uncropped versions of the whole DNA fingerprinteing patterns either for ISSR and SCoT molecular makrers are accompanied the manuscript as Supplementary Figs. 1 and 2, respectively

Photographs of some representative agarose gel electrophoresis of PCR fingerprinting in the 12 wheat cultivars are shown in Fig. 1A. Photographs of ISSR primers PCR fingerprinting are given as Supplementary Fig. S1. The polymorphism metrics (TNAs, MAs, PAs, %P, PIC, RP, and MI) of the 12 ISSR primers are summarized in Table 2A. All primers generated 150 amplicons, including 76 polymorphic amplicons (50.67%). The number of generated amplicons per primer varied from 8 for ISSR primer-12 to 23 for ISSR primer-13. The number of polymorphic markers also ranged from 1 for primer ISSR-12 to 12 and 15 for ISSR primer-9 and ISSR primer-13, respectively. The average number of polymorphic amplicons per-ISSR primer was 6.33. The PIC values for the ISSR primers ranged from 0.11 (the lowest value) for ISSR primer-12 to 0.36 for ISSR primer-13 (the highest value). The ISSR primer-9 revealed pronounced discrimination of 80% polymorphism. On the other hand, ISSR primer-12 recorded the lowest polymorphism of 13%. The ISSR primer-13 recorded the highest PIC and RP values of 0.36 and 9.56, respectively (Table 2A).

The SCoT fingerprinting profiles revealed by the 11 primers are illustrated in Fig. 1B, and the ScoT fingerprinting produced by the 11 primers is provided as Supplementary Fig. S2. The polymorphism metrics (TNAs, MAs, PAs, %P, PIC, RP, and MI) of the 11 ScoT primers are summarized in Table 2B. A total of 153 amplicons were produced, including 85 polymorphic (55.56%) markers. The total number of amplified PCR amplicons varied between 10 for SCoT primer-10 and 24 for SCoT primer-5, and the number of polymorphic bands also ranged from 1 for SCoT primer-28 to 21 for SCoT primer-5. The average number of polymorphic bands was 7.7 per primer. The PIC values range from 0.17 for SCoT primer-28 to 0.37 for SCoT primer-5 and primer-20. Also, the SCoT primer-5 revealed a pronounced polymorphism of 88% and recorded the highest RP of 10.67.

### Genetic relationships of wheat cultivars as revealed by ISSR and SCoT markers

**Fig. 2:**
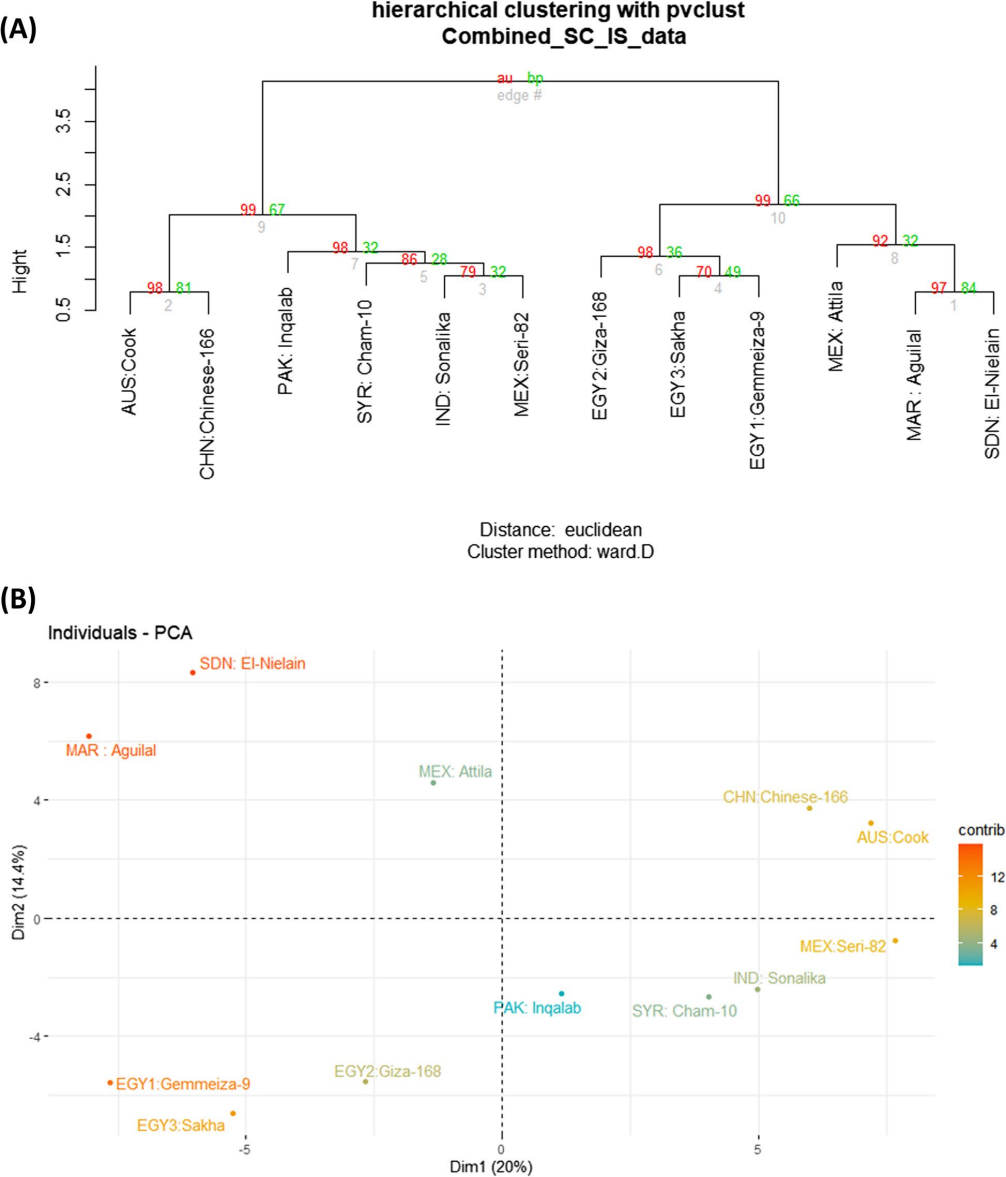
**(A)** Euclidean distance cluster tree and (**B**) PCA scatter diagram illustrating the genetic diversity between the 12 wheat cultivars based on the analysis of ISSR and SCoT markers polymorphism using the "pvclust" R package in R software

The ISSR and SCoT markers binary data, scored 1 for presence and 0 for absence, for the 12 wheat cultivars, were used to construct an Euclidean distance tree (Fig. 2A). The tree shows the differentiation of the 12 cultivars into two main groups. In group I, the three Egyptian cultivars EGY2: Giza-168, EGY1: Gemmeiza-9, and EGY3: Sakha-93 are grouped as a cluster, with the three cultivars MEX: Attila, MAR: Aguilal, and SDN: El-Nielain as the second cluster. In group II, the two cultivars: AUS: Cook and CHN: Chinese-166, are recognized as a cluster from another cluster, comprising the cultivars MEX: Seri-82, IND: Sonalika, SYR: Cham-10, and PAK: Inqalab-91. The differentiation of the examined wheat cultivars described in Fig. 2A is supported by two additional cluster trees that were constructed based on the recorded data of ISSR and SCoT markers, as two independent analyses, using the Dice's coefficient application of PAST software (Supplementary Materials, Fig. S3A, B). In both trees, the 12 wheat cultivars were generally differentiated as in the tree based on the ISSR, and SCoT markers combined analysis shown in Fig. 2A.

The PCA scatter diagram of 12 wheat cultivars based on the ISSR and SCoT markers combined by plotting Dim1 (20%) and Dim2 (14%) (Fig. 2B), which agrees with the clustering analysis (Fig. 2A). The three Egyptian cultivars EGY1: Gemmeiza-9, EGY2: Giza-168, and EGY3: Sakha-93 are grouped closely together as in the cluster tree shown in Fig. 2A but are distinguished from the three cultivars MEX: Attila, MAR: Aguilal, and SDN: El-Nielain, as grouped in the cluster tree of Fig. 2A. On the other hand, the two cultivars AUS: Cook and CHN: Chinese-166 are plotted close to the cultivars MEX: Seri-82 and IND: Sonalika and the cultivars SYR: Cham-10 and PAK: Inqalab-91, which formed two clusters of group II, as illustrated in Fig. 2A.

### Relationship of wheat cultivars based on *rbcL* and *matK* barcoding

The amplified DNA fragments of the *rbc*L and *mat*K genes recorded 600 bps and 800 bps, respectively, as shown in Supplementary Materials, Fig. S4 and Table 3. To affirm that the *rbc*L and *mat*K sequences presented in this study belonged to *T. aestivum*, a BLASTN nucleotide to nucleotide function analysis indicated that all the sequences matched *rbc*L and *mat*K sequences belonging to *T. aestivum* accessions in the NCBI GenBank. Additional information concerning the estimates of sequence(s) variation of *rbc*L and *mat*K barcoding loci, particularly summarized PCR amplification results, sequencing success, variability, the aligned length, variable sites and their proportion, and statistical simulation of BLAST Sequence homology of wheat cultivars for barcoding the *rbc*L and *mat*K genes is given in Supplementary Table S1. Pairwise distances were analyzed from the conserved *rbc*L and *mat*K sequences.

**Fig. 3:**
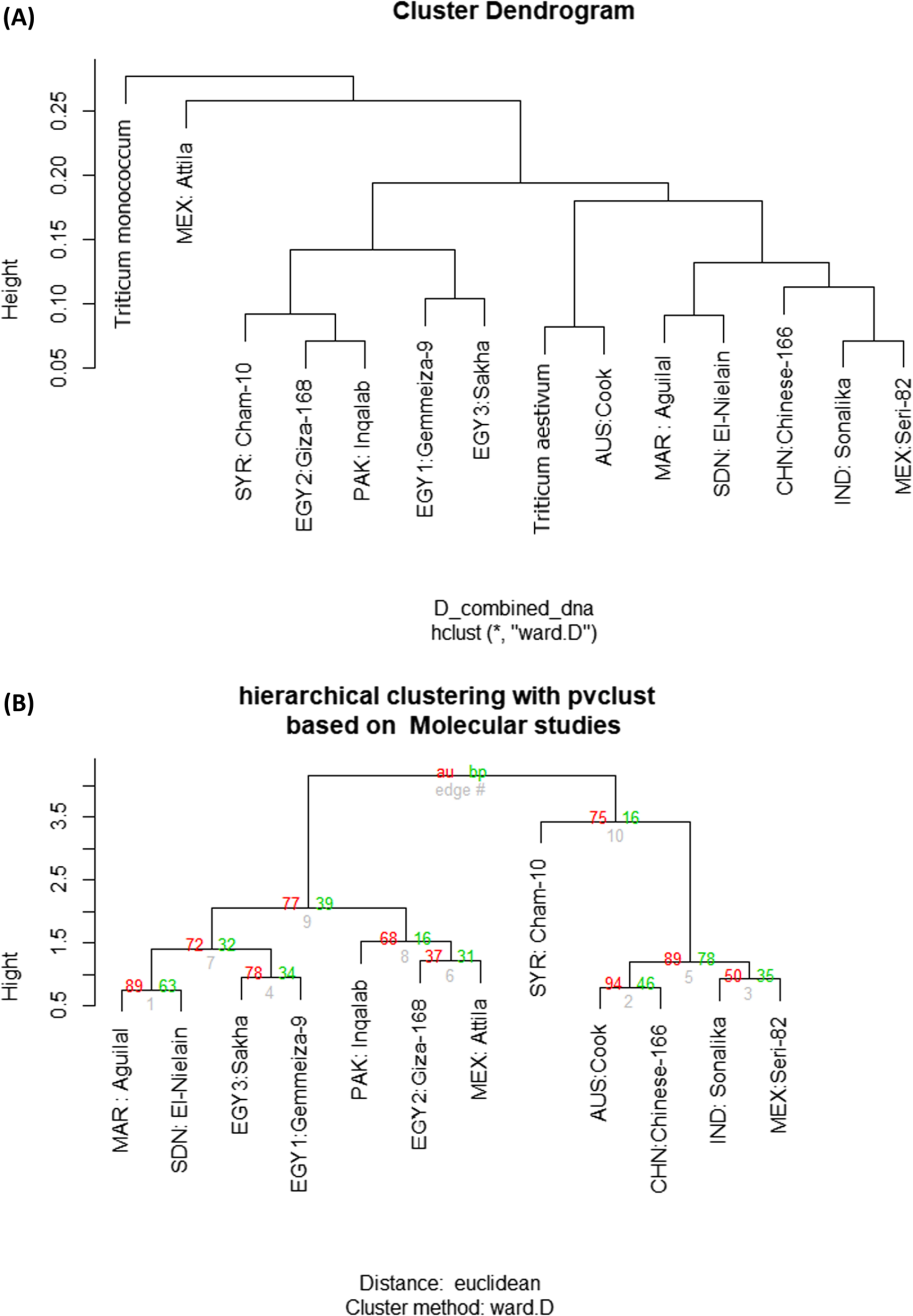
**(A)** Cluster tree constructed using R software illustrating genetic relatedness among the 12 wheat cultivars as revealed by sequence variation of combined *rbc*L (*T. monococcum* accession [KX282834.1] and *T. aestivum* accession [KR092108.1]) and *mat*K (*T. aestivum* accession [AF164405.1], *T. monococcum* [HM540031.1]) which were used as reference sequences. **(B**) Euclidean distance tree, constructed using the "pvclust" R package in R-Software, illustrating the genetic diversity among the wheat cultivars, as revealed by the analysis of the ISSR and SCoT markers polymorphism and DNA barcoding of *rbc*L and *mat*K sequence variation

The *rbc*L and *mat*K sequence variations were combined to construct a cluster tree illustrating genetic relatedness among the studied wheat cultivars using two NCBI-extracted *rbc*L sequences of *T. aestivum* accession [KR092108.1] and *Triticum monococcum* accession [KX282834.1]. In this tree (Fig. 3A), *T. monococcum* accession [KX282834.1] and the cv. MEX: Attila, a North American cultivar, were differentiated into two branches, and the other cultivars were distinguished into two groups. In group I, the Egyptian cultivars; EGY1: Gemmeiza-9 and EGY3: Sakha-93 were grouped as one branch, and the cultivar EGY2: Giza-168, cultivar PAK: Inqalab-91, and cultivar SYR: Cham-10 cultivars were grouped into a second branch. In group II, the reference *T. aestivum* accession [KR092108.1] and the AUS: Cook was differentiated from the other five cultivars into two separate branches, one for MAR: Aguilal and the SDN: El-Nielain cultivars, while the other one is for CHN: Chinese-166, MEX: Seri-82, and IND: Sonalika cultivars (Fig. 3A). Additional independent trees based on *rbc*L and *mat*K sequence variation analysis using *T.* *aestivum* accession and *T. monococcum* accession as outgroups are provided (Supplementary Materials, Fig. S5A, B). The differentiation of the examined wheat cultivars in that table is comparable to their differentiation as presented in the tree shown in Fig. 3A.

### Relationships between wheat cultivars based on genomic DNA fingerprinting and barcoding outcomes

An Euclidean distance tree illustrates the genetic diversity among the studied wheat cultivars, as revealed by analysis of ISSR and SCoT fingerprinting combined with the *rbc*L and *mat*K sequences (Fig. 3B), divided the 12 wheat cultivars into two major groups. Group I includes two clusters; one consists of the two Egyptian cultivars EGY1: Gemmeiza-9, EGY3: Sakha-93, MAR: Aguilal, and SDN: El-Nielain, while the other includes EGY2: Giza-168, MEX: Attila, and PAK: Inqalab-91. Group II consists of the SYR: Cham-10, as a distinct branch, and the cultivars MEX: Seri-82, CHN: Chinese-166, IND: Sonalika, and AUS: Cook.

On the other hand, in a tree based on *rbc*L sequence variation (Supplementary Materials, Fig. S5A), *T. monococcum* accession [KX282834.1] was isolated from all other cultivars, while MAR: Aguilal and MEX: Attila cultivars, as well as the two Egyptian ones; EGY1: Gemmeiza-9 and EGY3: Sakha-93, are clustered as two distinct branches of the tree. The remaining nine cultivars represented a major group, but each cultivar represented a single line (Fig. S5A).

On the other hand, in a tree based on the analysis of *mat*K sequences, the *T. aestivum* accession [AF164405.1], *T. monococcum* [HM540031.1], and the Australian cultivar AUS: Cook was isolated as a separate cluster from two major clusters representing the remaining wheat cultivars; the first cluster comprised the Egyptian cultivars together with MEX: Attila and PAK: Inqalab-91, while the second one includes the remaining wheat cultivars (Supplementary Materials, Fig. S5B). A PCA scatter diagram of the 12 wheat cultivars (Supplementary Materials, Fig. S6) reflected the differentiation among the examined cultivars, as illustrated in the combined tree shown in Fig. 3B.

### Relationships between wheat cultivars based on the grain surface sculpture

**Fig. 4:**
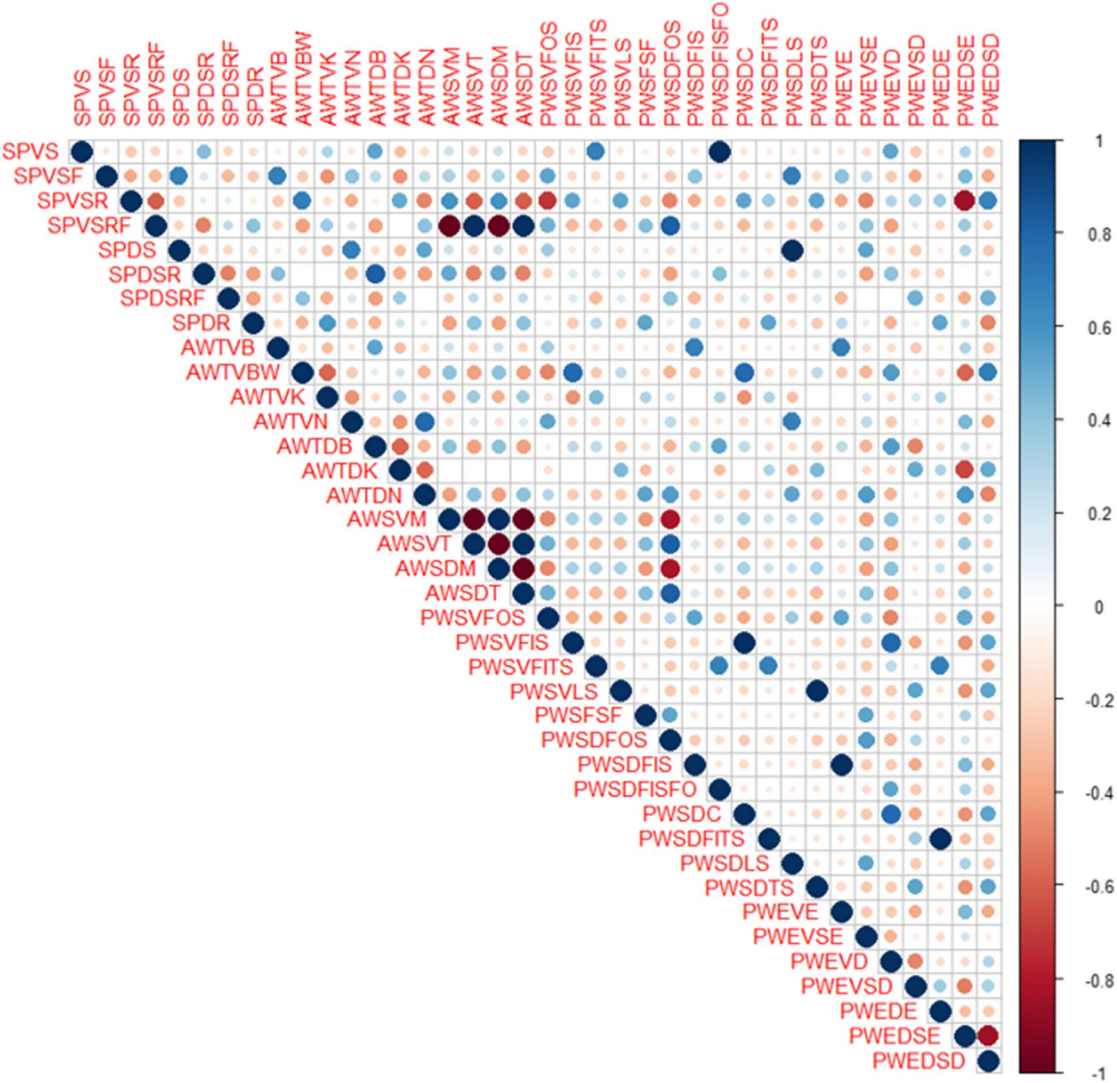
Correlogram was based on the correlation coefficients of 38 macro-morphological features in 12 wheat cultivars based on grain surface sculpture. Abbreviations of the macro-morphological features are listed in the Supplementary Table S2

The grain surface sculpture traits analysis revealed a somewhat different grouping of the examined cultivars but indicated a close similarity between cv. Cham-10 from Syria and the two Egyptian cultivars Gemmeiza-9 and Sakha-93. The grains sculpture revealed a wide range of variation in the ventral and dorsal sides of the grains of the examined cultivars (Supplementary Table S2). A total of 38 exomorphic character states of 10 main characters were scored in the studied cultivars (Supplementary Table S2). These states were then analyzed for their correlation with each other (Fig. 4). High correlations were recorded between AWSDT, AWSVT, and SPVSRF that might play a prominent role in distinguishing the two Egyptian cultivars, EGY1: Gemmeiza-9 and EGY3: Sakha-93 from EGY2: Giza-168. Also, AWSDM and AWSVM were correlated and negatively correlated to AWSDT and AWSVT.

**Fig. 5:**
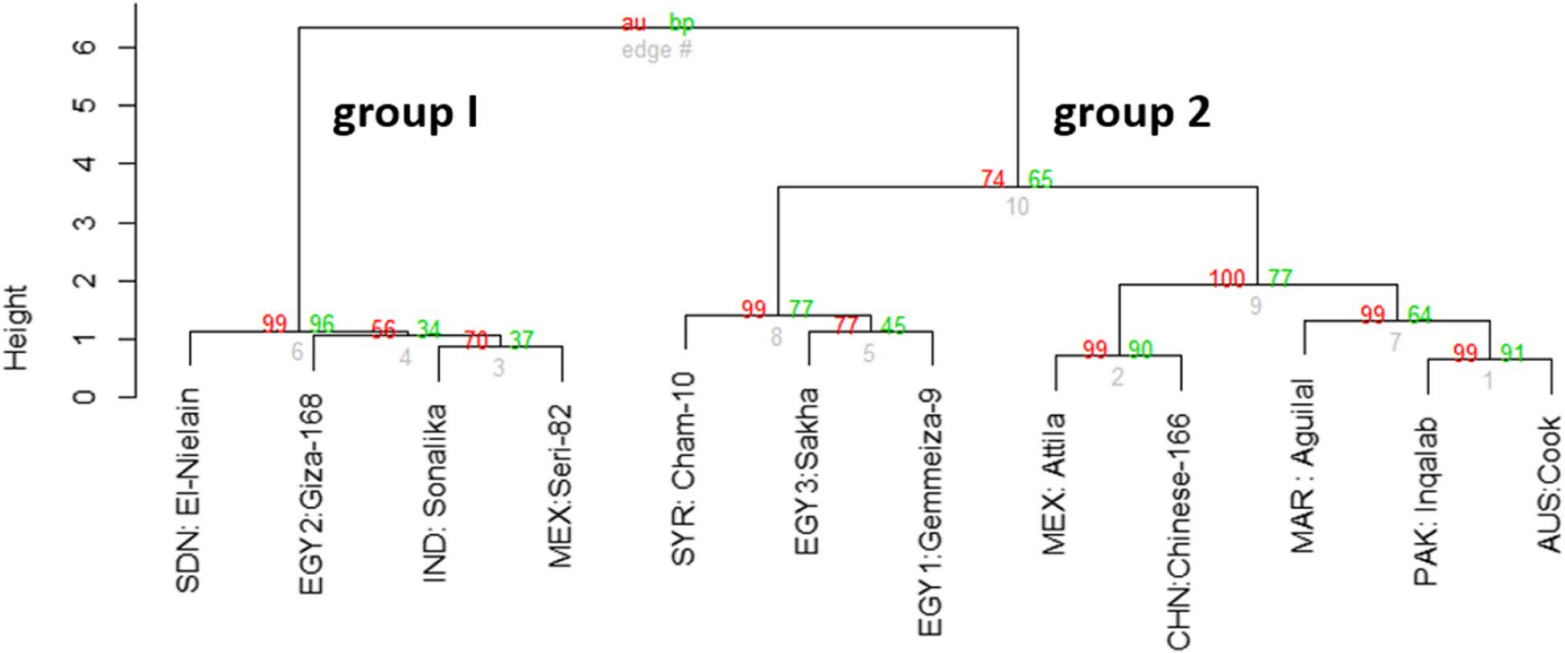
Distance UPGMA distance tree illustrating the genetic distance, based on the analysis of 38 grain surface sculpture traits of the twelve *T. aestivum* cultivars using R Software

A distance cluster tree, using the UPGMA algorithm, illustrating the classification of wheat cultivars based on the grain sculpture data is shown in Fig. 5. This tree divided the cultivars into two major groups. Group, I include the Egyptian cultivar EGY2: Giza-168, cultivars MEX: Seri-82, SDN: El-Nielain, and the IND: Sonalika. Group II, a larger group, includes the two Egyptian cultivars, EGY1: Gemmeiza-9 and EGY3: Sakha-93, grouped with cv. SYR: Cham-10, as a cluster, while the rest of the studied cultivars (cv. MEX: Attila and cv. CHN: Chinese-166, cv. MAR: Aguilal, cv. PAK: Inqalab-91 and cv. AUS: Cook. were grouped in another cluster (Fig. 5).

## Discussion

Genetic diversity was assessed using 303 polymorphic markers, including 151 ISSR markers produced by 12 ISSR and 155 SCoT markers produced by 11 SCoT primers, with a low average of 48.25% polymorphism and a high average of 13.3 bands per primer. It was also noticed that the number of MAs (monomorphic amplicons) was higher for ISSR markers than for SCoT markers. On the other hand, SCoT markers recorded a higher proportion of polymorphic amplicons (PAs) than ISSRs. Furthermore, SCoT markers also recorded higher PIC, RP, and MI mean values than ISSR markers. However, the highest RP value per primer was recorded for the ISSR-13 primer. Some Northern African wheat cultivars reported low levels of polymorphism ranging from 8 to 57% by the SCoT primers [28]. The low polymorphism percentage might be attributed to low genetic diversity and high conservation among the examined wheat cultivars. Genetic diversity and relationships among eight cultivars of Egyptian wheat, including cv. Sakha-93 and Giza-168 using six ISSR primers and eight SCoT primers, were investigated by Abdel-Lateif and Hewedy [45]. The ISSR primers produced 34 bands, including 23 (68%) polymorphic markers, with a mean of 4.6 per primer. These numbers are much lower than those recorded in the current study of 151 ISSR amplicons, including 76 (50.67%) polymorphic markers with an average of 6.33 per primer. Abdel-Lateif and Hewedy [45] also reported a lower number of SCoT markers (32 bands), including 19 (59%) PAs with a mean of 3.16, whereas 155 bands were amplified in the current study, including 84 ( 54.19%) PAs with an average of 7.63 per primer.

Multivariate clustering and PCA scatter plot of ISSR, and SCoT markers grouped the three Egyptian cultivars EGY1: Gemmeiza-9, EGY2: Giza-168, and EGY3: Sakha-93 together with a cv. El-Nielain from Sudan, cv. Aguilal from Morocco, and cv. Attila from Mexico. In a second group, ISSR and SCoT data analysis showed a close relationship between cv. Cook from Australia and the Chinese-166 cultivar differentiated from the other four cultivars; cv. Cham-10 from Syria, cv. Seri-82 from Mexico, cv. Inqalab-91 from Pakistan, and cv. Sonalika from India. The abundance of the ISSR and SCoT markers polymorphism and significant sequence variation support the use of these molecular markers extensively for DNA fingerprinting as a useful tool and straight-forward techniques in genetic diversity studies [22, 29, 46] and confirm that the characterization based on DNA polymorphism, i.e., molecular markers basis is more efficient, accurate, and justifying their wide use in genetic diversity assessment in the last two decades.

The ISSRs involve amplifying genomic segments flanked by inversely oriented sequences closely spaced by microsatellites [13], while the SCoT sequence is a short, conserved sequence surrounding the start codon ATG [14]. However, using the PAST software, both markers produced two similar classifications of the studied wheat cultivars expressed in constructing two similar trees. SCoT polymorphism analysis was performed to differentiate 14 wheat cultivars from North Africa (five of these cultivars were used in the current study). However, low levels of polymorphism ranging from 8 to 57%, with an average of 34.5%, were recorded in the 14 Northern African cultivars [28]. In the same context, Ibrahim et al. [29] reported polymorphism percentages ranging from 0 to 67%, with an average of 38.4% using 30 SCoT primers in 8 wheat cultivars from Asia. However, these levels were considered sufficient to indicate the cultivars' genetic differentiation and generate genomic loci that encode functional mRNA. In the current study, a higher level of polymorphism ranging between 11 and 88%, with an average of 51.4%, was recorded using 12 primers in the global 12 examined wheat cultivars. In this connection, about 923 ISSR and sequence-related amplified polymorphism (SRAP) molecular markers, besides numerous phenotypic traits were employed to monitor triggered improvement of orchadgrass polycross populations subjected to water deficit conditions [24, 25].

SCoTs markers and the gene/trait defining them to be directly employed in breeding programs than SSRs, ISSRs, and RAPDs in fingerprinting newly synthesized tritordeums and their respective parents [47]. In *Triticum urartu*, 72 accessions from Iran were grouped into two main clusters using two sets of markers. However, the grouping patterns were not obeyed by the geographic origins of the accessions [30]. The latter study also showed that Iranian *T. urartu*, especially the Kerend-e-Gharb and Sisakht-Pataveh populations, could greatly affect wheat improvement. Taheri et al. [48] also used IRAP and REMAP markers to assess the genetic divergence and relatedness among *T. urartu* and *T. boeoticum* populations in Iran. Cluster and PCA analyses using REMAP data grouped the populations based on the species and geographical origin. Although the grouping based on IRAP could not separate the two species, considerable diversity was observed among and within the studied populations based on both marker systems. In the same context, durum wheat genotypes were differentiated into five groups. The clustering of these genotypes based on the SCoT markers polymorphism supported the best clustering pattern and was more efficient, as reported by Etminan et al. [26]. Moreover, Ghobadi et al. [31] analyzed the genetic diversity and population structure in *T. aestivum*, *Aegilops cylindrica* and *Aegilops crassa* using CBDP and SCoT markers. They showed that both molecular markers grouped all samples based on their genomic constitutions and concluded that both techniques effectively evaluate the genetic diversity in wild relatives of wheat.

In the current study, the tree based on the *rbc*L and *mat*K sequence analysis differs mainly from the tree based on the SCoT and ISSR data, particularly the close relation of cv. EGY2: Giza-168, cv. PAK: Inqalab-91, and cv. SYR: Cham-10 in clade 1 and between cv. MAR: Aguilal and cv. SDN: El-Nielain with cultivars of cv. CHN: Chinese-166, cv. MEX: Seri-82, and cv. IND: Sonalika. In the tree based on the *rbc*L sequence variations, the cultivars were isolated as three groups with some resemblance to the trees based on the ISSR and SCoT fingerprinting polymorphism. In particular, the grouping of cv. MAR: Aguilal and cv. MEX: Attila and the two Egyptian cultivars EGY1: Gemmeiza-9 and EGY3: Sakha-93. On the other hand, the tree based on the analysis of *mat*K sequences alone clearly isolated *T. aestivum* accession [AF164405.1] and the Australian cv. AUS: Cook as a clade from another two major clades representing the remaining wheat cultivars; one clade of cv. EGY2: Giza-168 and cv. MEX: Attila together with a cv. EGY1: Gemmeiza-9, cv. EGY3: Sakha-93, and cv. PAK: Inqalab-91, while the other cultivars were grouped into another clade.

The *mat*K and *rbc*L DNA barcoding loci were used in the distinction between different Egyptian landraces of *T. aestivum* and *T. turgidum* subsp durum using eleven different landraces, and seven local varieties were examined for their ability to distinguish between other Poaceae crops and herbs, including *Avena fatua*, *Hordeum vulgare*, and *Hordeum apertum* [49]. The results showed that *mat*K and *rbc*L had a limited capability in differentiating between the questionable *Triticum* accessions. In the same study, the conducted in silico analysis emphasized the differentiation potential of using combinations of chloroplast intergenic regions more than coding genes in the ten *Triticum* species and sub-species [49].

In the presented study, the differences in the wheat cultivars genotypes based on SCoT and ISSR fingerprinting had great potential in differentiating *T. asetivum* cultivars other than *rbc*L and *mat*K sequence analysis. This may be attributed to the *rbc*L and *mat*K genes, similar to other chloroplast genes, which elucidate diversity at higher taxonomic levels [50, 51]. On the other hand, the ISSR markers, which amplify genomic segments flanked by inversely oriented sequences closely spaced by microsatellites [13] and the SCoT sequence, which amplifies the conserved sequence surrounding the start codon of functional genes [14], reveal sufficient polymorphism for stable and reproducible differentiation below the species level as reported here between the cultivars of wheat. Feltaous [52] reported that genetic diversity among Egyptian wheat cultivars showed a narrow morphological variation compared to the SSR markers polymorphism. The same study concluded that SSR markers were more accurate and informative than morphological characters. This result supports the use of DNA fingerprinting in estimating the genetic diversity of wheat cultivars, as molecular markers are abundant, easy to handle, and independent of environmental factors. Abdel-Lateif and Hewedy [45] and Badr et al. [53] presented results supporting our conclusion that SCoT and ISSR markers produced higher polymorphism than SCoT markers and can be employed in wheat breeding programs to evaluate genetic diversity that may be used in producing new cultivars of more resilient to the environmental changes in the future.

The grains surface sculpture using SEM screening revealed 38 exomorphic character states and offered various variations among the studied cultivars. The analysis of these traits indicated a close resemblance between cv. Cham-10 from Syria and the two Egyptian cultivars Gemmeiza-9 and Sakha-93. However, the grouping pattern obtained from the analysis of the grain surface sculpture traits revealed wasn't in accord with that obtained from the molecular studies of the examined wheat cultivars. The value of the features of the grain in the classification of grasses at the species level, mainly using grain surface scanning, is well documented [41, 43, 44].

## Conclusions

In conclusion, the differentiation and characterization of wheat cultivars using the ISSR and the SCoT markers using clustering and PCA analyses grouped the three Egyptian cultivars; Gemmeiza-9, Giza-168, and Sakha-93, together with cv. El-Nielain from Sudan, cv. Aguilal from Morocco, and cv. Attila from Mexico in one group, and the other cultivars in a second group. The close relationship between cv. Cook from Australia and cv. Chinese-166 differentiated from the other four cultivars from Syria, Mexico, Pakistan, and Bangladesh. The analysis of the chloroplast DNA agrees with the ISSR and the SCoT markers for some cultivars but differs for others, such as the close resemblance between cv. Cham-10 from Syria and the two Egyptian cultivars Gemmeiza-9 and Sakha-93. The ISSR and SCoT analyses significantly expressed the genetic diversity among the studied wheat cultivars with higher differentiation levels than the *rbc*L and *mat*K genes. The differentiation of the cultivars using the *rbc*L and *mat*K sequences variation and the SEM screening of the grain surface sculpture may add helpful insights into the cultivars' genetic diversity and provide essential knowledge for their selection as genetic resources in breeding new cultivars.

## Methods

### Plant material

The present study dealt with twelve selected cultivars of bread wheat (*Triticum aestivum* L.). The grains were received with a full identification file from the Libyan Agricultural Research Center, the eastern region sub-center in Elbeida/Benghazi, Libya, based on a joint collaboration with the Food and Agriculture Organization of the United Nations (FAO-Libya) and International Center for Agricultural Research in Dry Land (ICARDA), Aleppo, Syria. Affirmed needed permissions were obtained and complied with the International Union for Conservation of Nature (IUCN) approved by the 27^th^ meeting of the IUCN council, GLAND SWITZERLAND (1989). Dr. Mohamed Tantawy, professor of plant taxonomy and flora, Department of Botany, Faculty of Science, Ain Shams University, Cairo, Egypt, double checked these cultivars and the voucher specimens were kept in the Herbarium of Department of Botany, Ain Shams University (CAIA; http://sweetgum.nybg.org/science/ih/herbarium-details/?irn=123925). The country of origin, country code, and pedigree of the cultivars used are listed in Table 1. They include three cultivars from Egypt and one from nine other countries, each in Africa, Asia, Europe, Middle America, and Australia.

**Table 1:** Origin, codes, names, and pedigree of the wheat cultivars as recorded by ICARDA and the GenBank deposited accession numbers for the *rbc*L and *mat*K genes for the 12 cultivars of bread wheat

Serial No	Origin	Code^a^	Cultivar name and pedigree	Genebank Accession* rbc*L	Genebank Accession* mat*K
**1**	Egypt	EGY1	Gemmeiza-9 ARC (Ald”S”/Huac”S”//CMH74A.630/5 × CGM4583-5GM-1GM-0GM)	MT797209	MW620988
**2**	Egypt	EGY2	Giza-168 ARC (MIL/BUC//Seri CM93046-8 M-0Y-0 M-2Y-0B)	MT797200	MZ207916
**3**	Egypt	EGY3	Sakha-93 ARC (Sakha 92/TR 810328 S 8871-1S-2S-1S-0S)	MT797205	MW598256
**4**	Morocco	MAR	Aguilal (Saïs*2/1/KS-85–14-2)	MT797208	MW598252
**5**	Sudan	SDN	El-Nielain (S948.A1/7*SANTA ELENA, CMH 72A.390-OSDN)	MT797202	MW620987
**6**	India	IND	Sonalika (II53.388/AN//YT54/N10B/3/LR/ 4/B4946.A.4.18.2.1Y/Y53// 3*Y50)	MT797204	MW598251
**7**	China	CHN	Chinese 166 (S-Chinese 165(= Intro. from CHN); Chinese Land Variety [JIC])	MT797207	MW598250
**8**	Pakistan	PAK	Inqalab-91 (WL-711/Crow)	MT797201	MW598254
**9**	Syria	SYR	Cham-10 (Kauz//Kauz/Star)	MT797203	MW598255
**10**	Australia	AUS	Cook (Timgalen/ Condor's'//Condor)	MT797211	MW598257
**11**	Mexico	MEX	Seri-82 (Kavkaz/Buho sib//Kalyansona/Bluebird)	MT797206	MZ207917
**12**	Mexico	MEX	Attila (ND/VG9144//KAL/BB/3/YACO/ 3/VEERY #5)	MT797210	MW598253

^a^Three-digit codes used in this study are according to official ISO country codes listed in (http://www.nationsonline.org/oneworld/country_code_list.htm and http://www.worldatlas.com/aatlas/ctycodes.htm). All the listed cultivars were used for ISSR, SCoT molecular markers, and DNA barcoding of r*bc*L and *mat*K genes. The origin of the studied cultivars were listed according to United States Department of Agriculture (USDA) (https://www.usda.gov/)

### Extraction of genomic DNA

DNA extraction was performed from 50 mg freeze-dried powder of ground grains of the wheat cultivars using the DNeasy Plant Mini Kit (QIAGEN, Hilden, Germany). Extracted DNA was quantified as described by the Molecular Cloning Laboratory Manual [54]. The purity was measured using an ND-1000 spectrophotometer (Nano-Drop Technologies, Thermo Fisher Scientific Inc.).

### ISSR/SCoT primers and ISSR/SCoT PCR amplification

The 12 ISSR and 11 SCoT primers, used for DNA fingerprinting, were synthesized by the HVD Egypt under license from HVD Vertriebs-Ges. GmbH, Vienna, Austria, delivered, rehydrated, and stored at -20 °C. The PCR technique for ISSR and SCoT was carried out, as described in Badr et al. [17], in a 25 μl reaction volume containing 1X PCR buffer, 1.5 mM MgCl_2_, 0.15 mM dNTPs, 25 µM primer, 25 ng wheat DNA, and 1 unit of Phusion^®^ High-Fidelity DNA Polymerase (Espoo, Finland). PCR was performed using a PerkinElmer GeneAmp^®^ PCR System 9700 (PE Applied Biosystems, Bedford, MA, United States). The ISSR and SCoT primers; name, sequence, GC%, TM°C, as well as the information on the amplicons per primer, in the 12 wheat cultivars, are given in Table 2A and B, respectively.

**Table 2:** List of the ISSR (A) and SCoT (B) primers; name, sequence, GC%, TM°C, total number of amplicons (TNAs) per primer, monomorphic amplicons (MAs), polymorphic amplicons (PAs), percentage of polymorphism (%P), polymorphic information content (PIC), resolving power (RP), and marker index (MI) as revealed by ISSR and SCoT profiles in the 12 wheat cultivars. The primer sequences were synthesized by the HVD Egypt company

**(A) ISSR Primer list**									
**Ser**	**Primer Name**	**Sequence (5́ › 3́)**	**TNAs**	**MAs**	**PAs**	**% P**	**PIC**	**RP**	**MI**
**1**	ISSR-1	AGAGAGAGAGAGAGAGYC	13	7	6	46%	0.34	7.69	0.023
**2**	ISSR-2	AGAGAGAGAGAGAGAGYG	11	7	4	36%	0.32	6.55	0.026
**3**	ISSR-6	CGCGATAGATAGATAGATA	14	5	9	64%	0.35	7.00	0.021
**4**	ISSR-7	GACGATAGATAGATAGATA	12	5	7	58%	0.33	6.67	0.025
**5**	ISSR-8	AGACAGACAGACAGACGC	9	6	3	33%	0.20	3.11	0.022
**6**	ISSR-9	GATAGATAGATAGATAGC	13	3	10	**77%**	0.32	5.54	0.022
**7**	ISSR-12	ACACACACACACACACYC	8	7	1	13%	0.11	1.50	0.013
**8**	ISSR-13	AGAGAGAGAGAGAGAGYT	26	9	17	65%	**0.37**	**10.31**	0.011
**9**	ISSR-14	CTCCTCCTCCTCCTCTT	11	7	4	36%	0.28	5.09	0.023
**10**	ISSR-15	CTCTCTCTCTCTCTCTRG	11	5	6	54%	0.26	4.54	0.023
**11**	ISSR-19	HVHTCCTCCTCCTCCTCC	13	7	6	46%	0.30	5..84	0.021
**12**	ISSR-20	HVHTGTGTGTGTGTGTGT	10	7	3	30%	0.21	3.40	0.118
**Total**			**151**	**75**	**76**	**-**	**-**	**-**	**-**
**Mean**					**6.33**	**46.5**	**0.28**	**5.12**	**0.028**
**(B) SCoT Primer list**									
**Ser**	**Primer Name**	**Sequence (5́ › 3́)**	**TNAs**	**MAs**	**PAs**	**% P**	**PIC**	**RP**	**MI**
**1**	ScoT-2	ACCATGGCTACCACCGGC	18	9	9	50%	0.34	7.89	0.016
**2**	SCoT-3	ACGACATGGCGACCCACA	16	7	9	56%	0.33	6.75	0.018
**3**	SCoT-4	ACCATGGCTACCACCGCA	12	8	4	33%	0.19	3.00	0.015
**4**	SCoT-5	CAATGGCTACCACTAGCG	27	5	22	**81%**	**0.37**	**10.00**	0.009
**5**	SCoT-11	ACAATGGCTACCACTACC	11	5	6	55%	0.32	6.55	0.026
**6**	SCoT-12	CAACAATGGCTACCACCG	10	6	4	40%	0.32	6.60	0.028
**7**	SCoT-13	ACCATGGCTACCACGGCA	15	6	9	60%	0.27	4.93	0.017
**8**	SCoT-16	CCATGGCTACCACCGGCA	14	6	8	57%	0.32	6.71	0.021
**9**	SCoT-20	CAACAATGGCTACCACGC	11	4	7	64%	**0.37**	9.45	0.025
**10**	SCoT-22	CCATGGCTACCACCGCAC	12	7	5	42%	0.25	4.16	0.019
**11**	SCoT-28	CAACAATGGCTACCACCA	9	8	1	11%	0.17	2.44	0.018
**Total**			**155**	**71**	**84**	**-**	**-**	**-**	**-**
**Mean**					**7.6**	**50**	**0.30**	**6.23**	**0.019**

The amplification of ISSR markers was performed in 40 cycles as follows: an initial denaturation cycle at 94°C for 1 min, annealing at 50°C for 1 min, elongation at 72°C for 2 min, and a final extension for 5 min. On the other hand, SCoT amplification was performed in 35 cycles as follows: 5 min at 94°C denaturation, 7 min annealing at 50°C, and elongation in the final cycle at 72°C. The PCR products of ISSR and SCoT markers were separated on 1.5% agarose gel. Gels were stained with 100 µM/L EtBr (100 µM/L, Sigma‒Aldrich^®^) in 1X TBE. The PCR products were visualized and documented using a Bio-Rad ChemiDoc™ MP gel documentation and imaging system (Cat. no. 1708280).

### *rbc*L and  *mat*K chloroplast gene barcoding

The forward and reverse primer sequences used for barcoding are given in Table 3. The PCR amplification of the *rbc*L and *mat*K genes was performed at an initial denaturation for 5 min at 94°C followed by 40 cycles, each comprised of denaturation at 94°C for 30 s, annealing at 45°C for 30 s, and elongation at 72°C for 30 s. The primer extension was extended for 7 min at 72°C in the final cycle. PCR-specific products were subsequently electrophoresed in 1.5% (W/V) agarose, stained with 100 µM/L EtBr (Sigma-Aldrich^®^) in 1X TBE buffer, visualized, and finally documented according to the Molecular Cloning Laboratory Manual [54]. PCR-specific amplified fragments of *rbc*L and *mat*K were purified from agarose gel by QIAquik^®^ PCR PURIFICATION KIT (Qiagen Inc., Cat. no. 28106). Specific purified amplicons were then cloned, and the DNA sequencing protocol for the *rbc*L and *mat*K amplified fragments was executed as previously described by Badr et al. [17].

**Table 3:** Primer codes and sequences for barcoding the *rbc*L and *mat*K genes and their size in bps

Primer Code	Sequence	Product Size	Reference
***rbc*La-1F**	5’-ATGTCACCACAAACAGAGACTAAAGC-3’	600 bp	Fay et al. [55]
***rbc*L724-R**	5’-TCGCATGTACCTGCAGTAGC-3’		
***mat*K-472F**	5’-CCCRTYCATCTGGAAATCTTGGTTC-3’	700–800 bp	Yu et al. [56]
***mat*K-1248R**	5’-GCTRTRATAATGAGAAAGATTTCTGC-3’		

### Dissection of grain surface sculpture and grain traits used

The scanning electron microscope (SEM) grain surface sculpture was done at the Regional Center of Mycology and Biotechnology, Al-Azhar University, Cairo, Egypt. The procedures of sample mounting, coating with a gold sputter coater (SPI module), and examination by JEOL-JSM 5600 LV SEM were performed as described by Mohamed et al. and Ibrahim et al. [26, 27]. The grain sculpture was characterized by examining 5–10 grains of each cultivar following the scheme adopted by Murley [57]. The characteristics scored were combined to assess the genetic variability of the wide range of wheat genotypic sources. Extracted exomorphic characters and used abbreviations of analyzed sculptures are listed in Supplementary Table S1.

### Data analysis

Sharp, evident reproducible bands amplified as ISSR or SCoT markers in the agarose gel were scored as "1" for the presence and "0" for the absence. Differentiating between and resolving studied genotypes, the capacity of ISSR or SCoT primers was judged by calculating the polymorphic metrics summarized in Table 2 (TNAs, MAs, PAs, %P, PIC, RP, and MI). The PIC value for each primer was calculated according to Ghislain et al. [58]. The *rbc*L and *mat*K barcoding gene sequences were analyzed using Bayesian analysis with MrBayes software ver. 3.2 [59]. The best-fit substitution model (SYM + G) was chosen based on the Akaike information criterion inferred by MrModel-test v.2.3 [60]. The Markov chain Monte Carlo (MCMC) process was run for 1,000,000 generations, and the resulting trees were sampled every 1000 generations with 16 chains. Stationarity was accomplished when "the average standard deviation of split frequencies" remained < 0.01. The first 25% of runs were discarded as a relative burning. In the R-studio interface [61] to run R software, several packages "*seqinr*", "*adegenet*", "*DECIPHER*", and  "*ape*" were used, followed by reading the aligned data from the fasta file and creating a distance matrix for the alignment. The complete linkage method was used for the UPGMA dendrogram construction [62]. The phylogenetic correlation matrix corresponded to a given phylogenetic tree generated using MrBayes software [62]. Moreover, "*ggplot2*" packages was used to visualize the similarity and dissimilarity within and among the cultivars [63, 64].

For phenetic analysis, a data matrix for *T. aestivum* L. cultivars based on ten characters of grain surface sculpture for 38 characters' states was conducted (Supplementary Table S1). The phenetic binary data matrix was employed for grain surface sculpture traits. Hierarchical clustering (UPGMA) was used to determine how closely the species or varieties are related [65]. Euclidian distance has been used after the data matrix scaling and standardization [66]. Using "*pvclust*" R-package, the agglomerative cluster analysis was created [67]. The Principal Component Analysis (PCA) ordination analyses were employed to examine the repeatability of the grouping acquired by the cluster analyses (61). The R-packages "*factoextra*" and "*ggplot2*" were handled for visualizing the distance matrices "fviz_pca" that provide ggplot2- based innovative visualization of PCA [66]. Using the "Corrplot" package, the correlation coefficients for the variable's relationship were performed and visualized according to [68]. All previous packages were employed to run R software [69]. 

## Supplementary Information

**Additional file 1.**

**Additional file 2.**

**Additional file 3.**

## Data Availability

The *rbc*L and *mat*K sequences investigated in this study have been deposited in NCBI"GenBank" (https://www.ncbi.nlm.nih.gov/genbank/) with primary accession codes [the list of deposited accession codes with links, e.g., "MT797209; https://www.ncbi.nlm.nih.gov/nuccore/MT797209" was fully investigated in Table 1. Furthermore, all generated and/or analyzed datasets (e.g., binary matrices and multistate characteristics of grain surface sculpture) during this study were completely included within the manuscript's main text and its accompanied supplementary information.
