# Supplementary material for: Genetic diversity analysis in wheat cultivars using SCoT and ISSR markers, chloroplast DNA barcoding and grain SEM
Source: BMC Plant Biol. 2023 Apr 11;23:193. doi: 10.1186/s12870-023-04196-w (PMC10088244; doi:10.1186/s12870-023-04196-w)
Supplement: Supplementary file 1 — Additional file 1. [file 12870_2023_4196_MOESM1_ESM.docx]

**Supplementary Tables and Figures**

Genetic diversity analysis in wheat cultivars using SCoT and ISSR markers, chloroplast DNA barcoding and grain SEM

Heba H. Abouseada ^1^, Al-Safa H. Mohamed^1^, Samir S. Teleb^2^, Abdelfattah Badr^3^, Mohamed E. Tantawy^1^, Shafik D. Ibrahim^4^, Faten Y. Ellmouni^5^*, Mohamed Ibrahim^1^*

^1^ Department of Botany, Faculty of Science, Ain Shams University, Cairo, Egypt

^2^ Botany and Microbiology Department, Faculty of Science, Zagazig University, 44519, Zagazig, Sharqia, Egypt

^3^ Botany and Microbiology Department, Faculty of Science, Helwan University, Cairo, Egypt

^4^Agricultural Genetic Engineering Research Institute (AGERI), Agricultural Research Center (ARC), Giza, Egypt.

^5^ Botany and Microbiology Department, Faculty of Science, Fayoum University, Fayoum 63514, Egypt

*Correspondence: [fyl00@fayoum.edu.eg](mailto:fyl00@fayoum.edu.eg) (F.Y.E.) ORCID no. 0000-0002-9463-0008;

[m.shehata@sci.asu.edu.eg](mailto:m.shehata@sci.asu.edu.eg) (M.I.) ORCID no. 0000-0002-5401-5115

**Supplementary Table S1** Additional information concerning the estimates of sequence variation of *rbc*L and *mat*K barcoding loci, particularly summarized PCR amplification results, sequencing success, variability, the aligned length, variable sites and its proportion, and statistical simulation of BLAST Sequence homology of wheat cultivars for barcoding the *rbc*L and *mat*K genes.

| **Evaluation of two DNA barcode regions.** | **DNA barcode regions** | | | **Used software** |
| --- | --- | --- | --- | --- |
|  | ***mat*k** | ***rbc*L** | ***mat*k+ *rbc*L** |  |
| **Number of individuals** | 12 | 12 | - |  |
| **PCR success (%)** | 100 | 100 | - |  |
| **Sequence length** | 501 | 548 | 1031 |  |
| **Aligned length** | 501 | 548 | 1031 |  |
| **Number of variable sites** | 28/501 | 49/548 | 68/1031 | Analyzed by Mega X |
| **Number of indels** | 0 | 0 | 0 |  |
| **Number of informative sites (Parsim-info)** | 16/501 | 1/548 | 17/1031 |  |
| **Conserved regions** | 473/501 | 487/548 | 954/1031 |  |
| **Singleton** | 12/501 | 46/548 | 51/1031 |  |
| **Zero-fold** | 318/501 | 356/548 | 637/1031 |  |
| **Two-fold** | 116/501 | 88/548 | 221/1031 |  |
| **Four-fold** | 51/501 | 76/548 | 127/1031 |  |
| **Coverage (100%)** | 485/501 | 520/548 | 629/1031 |  |
| **CpG (Percent Conservation)** | 99/501 | 53/548 | 151/1031 |  |
| **Mean of distance** | 1.655131 | 0.419 | 1.374481 | Analyzed by R software |
| **median** | 1.66367 | 0 | 1.447121 |  |
| **max** | 3.363485 | 2.162399 | 2.59434 |  |
| **min** | 0 | 0 | 0 |  |
| **Base composition: A** | 0.307 | 0.267 | 0.287 |  |
| **Base composition: C** | 0.175 | 0.208 | 0.192 |  |
| **Base composition: G** | 0.135 | 0.235 | 0.183 |  |
| **Base composition: T** | 0.383 | 0.29 | 0.338 |  |
| **Total** | 6.01 kb | 6.58 kb | 12.37 kb |  |
| **GC content** | 31% | 44.30% | 37.5 |  |

| **Supplementary Table S2** List of extracted exomorphic characters, character states, and abbreviations as revealed by SEM as previously described by Mohamed et al. [28] and Ibrahim et al. [29]. | | |
| --- | --- | --- |
| **Character** | **Character state** | **Abbreviation** |
| 1. **Surface pattern-Ventral** | 1. Scalariform | SPVS |
|  | 1. Scalariform, foviate | SPVSF |
|  | 1. Salariform, reticulate | SPVSR |
|  | 1. Scalariform, reticulate, foveate. | SPVSRF |
| 1. **Surface pattern-Dorsal** | 1. Scalariform | SPDS |
|  | 1. Scalariform, reticulate | SPDSR |
|  | 1. Scalariform, reticulate, foveate. | SPDSRF |
|  | 1. reticulate | SPDR |
| 1. **Anticlinal wall-Thickness-ventral** | 1. Broad | AWTVB |
|  | 1. Broad *Epicuticular wax | AWTVBW |
|  | 1. Thick | AWTVK |
|  | 1. Thin | AWTVN |
| 1. **Anticlinal wall-Thickness-dorsal** | 1. Broad | AWTDB |
|  | 1. Thick | AWTDK |
|  | 1. Thin | AWTDN |
| 1. **Anticlinal wall-Surface-ventral** | 1. Smooth | AWSVM |
|  | 1. Striated | AWSVT |
| 1. **Anticlinal wall-Surface-dorsal** | 1. Smooth | AWSDM |
|  | 1. Striated | AWSDT |
| 1. **Periclinal wall-Surface- ventral** | 1. Foveate stria | PWSVFOS |
|  | 1. Fine stria | PWSVFIS |
|  | 1. Fine transverse stria | PWSVFITS |
|  | 1. Longitudinal Stria | PWSVLS |
|  | 1. Fine striated foveate | PWSFSF |
| 1. **Periclinal wall-Surface- dorsal** | 1. foveate stria | PWSDFOS |
|  | 1. Fine stria | PWSDFIS |
|  | 1. Fine stria foveate | PWSDFISFO |
|  | 1. Constricted | PWSDC |
|  | 1. Fine transverse stria | PWSDFITS |
|  | 1. Longitudinal Stria | PWSDLS |
|  | 1. Transverse Stria | PWSDTS |
| 1. **Periclinal wall-Elevation-ventral** | 1. Elevated | PWEVE |
|  | 1. Shallow elevated | PWEVSE |
|  | 1. Depressed | PWEVD |
|  | 1. Shallow depressed | PWEVSD |
| 1. **Periclinal wall-Elevation-dorsal** | 1. Elevated | PWEDE |
|  | 1. Shallow elevated | PWEDSE |
|  | 1. Shallow depressed | PWEDSD |

**Fig. S1. (ISSR fully uncropped figures)**


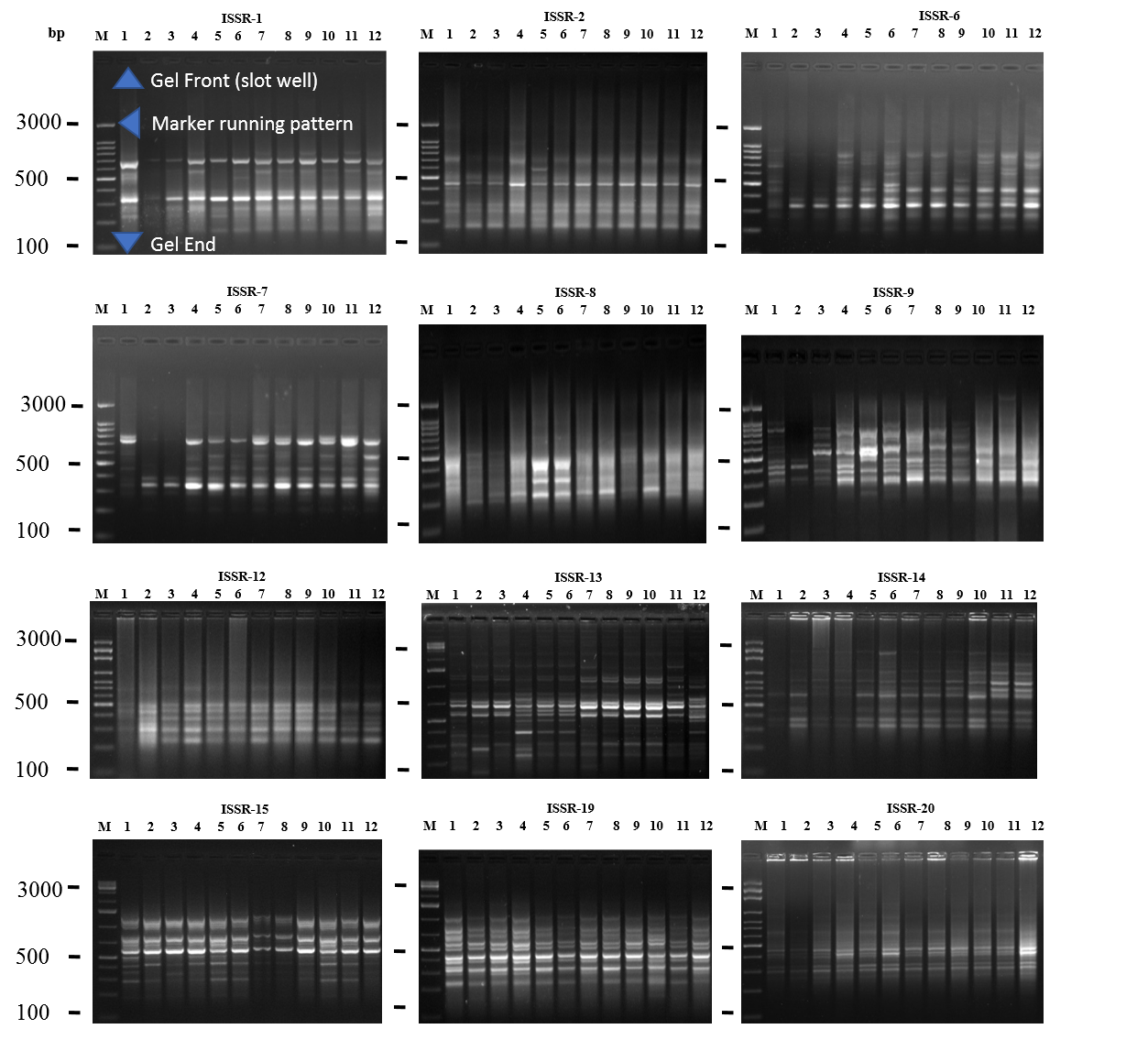


**Supplementary Fig. S1** Agarose gel electrophoresis of PCR amplicons of the eleven representative ISSR primers showing polymorphisms of ISSR markers. The DNA size marker (Cat. No. SB_07-11-0000S, MEDIBENA Life Science & Diagnostic Solutions, Vienna, Austria) (lane M) was used as a molecular size standard in bps. Numbers from 1 to 12 refer to the sampling numbers of the studied cultivars. The primer codes are listed in Table 2A.


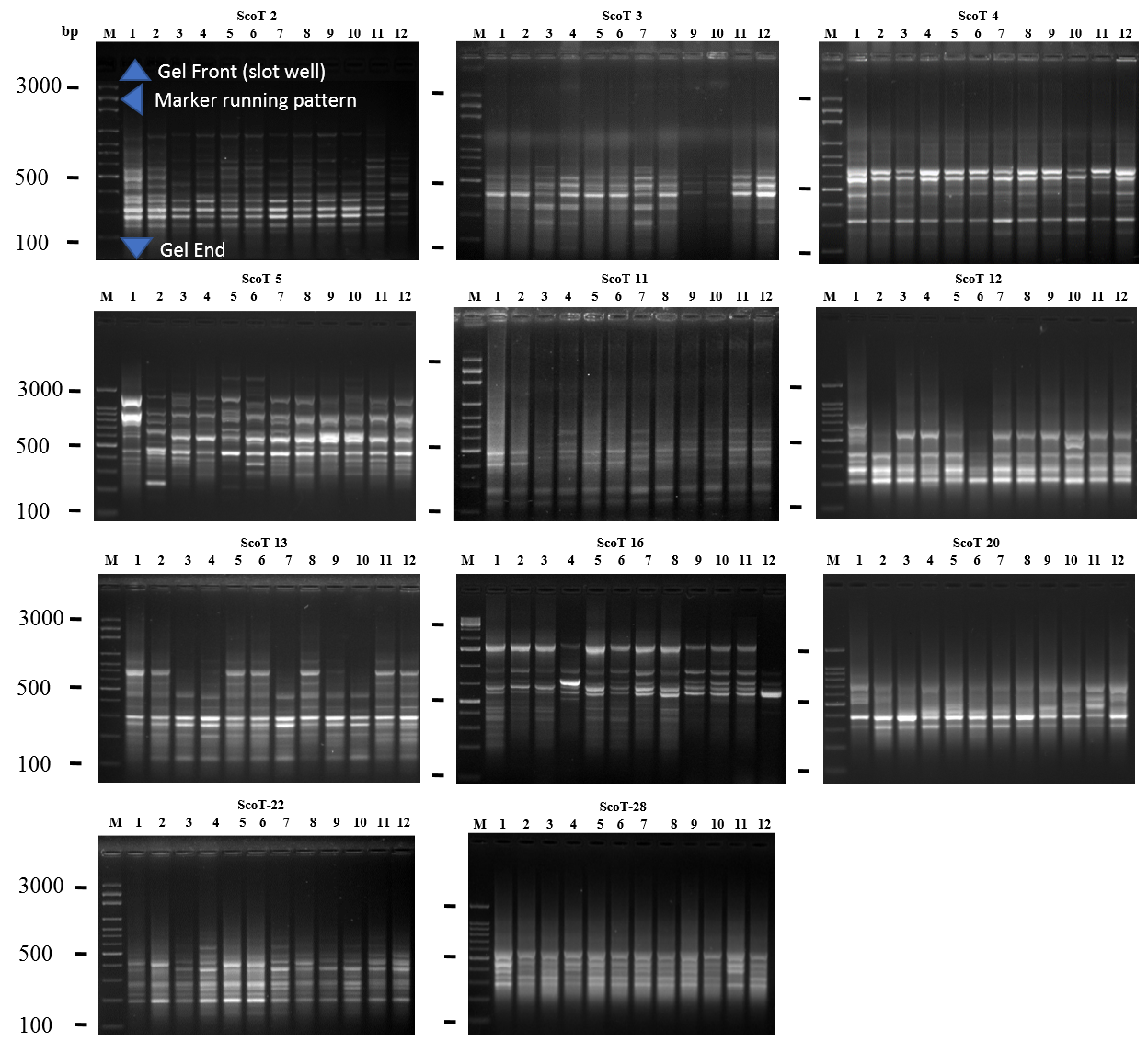
**Fig. S2. (SCoT fully uncropped figures)**

**Supplementary Fig. S2** Fully uncropped agarose gel electrophoresis of PCR amplicons of the eleven representative SCoT primers showing polymorphisms of SCoT markers. The DNA size marker (Cat. No. SB_07-11-0000S, MEDIBENA Life Science & Diagnostic Solutions, Vienna, Austria) (lane M) was used as a molecular size standard in bps. Numbers from 1 to 12 refer to the sampling numbers of the studied cultivars. The primer codes are listed in Table 2B.

**Fig. S3.**


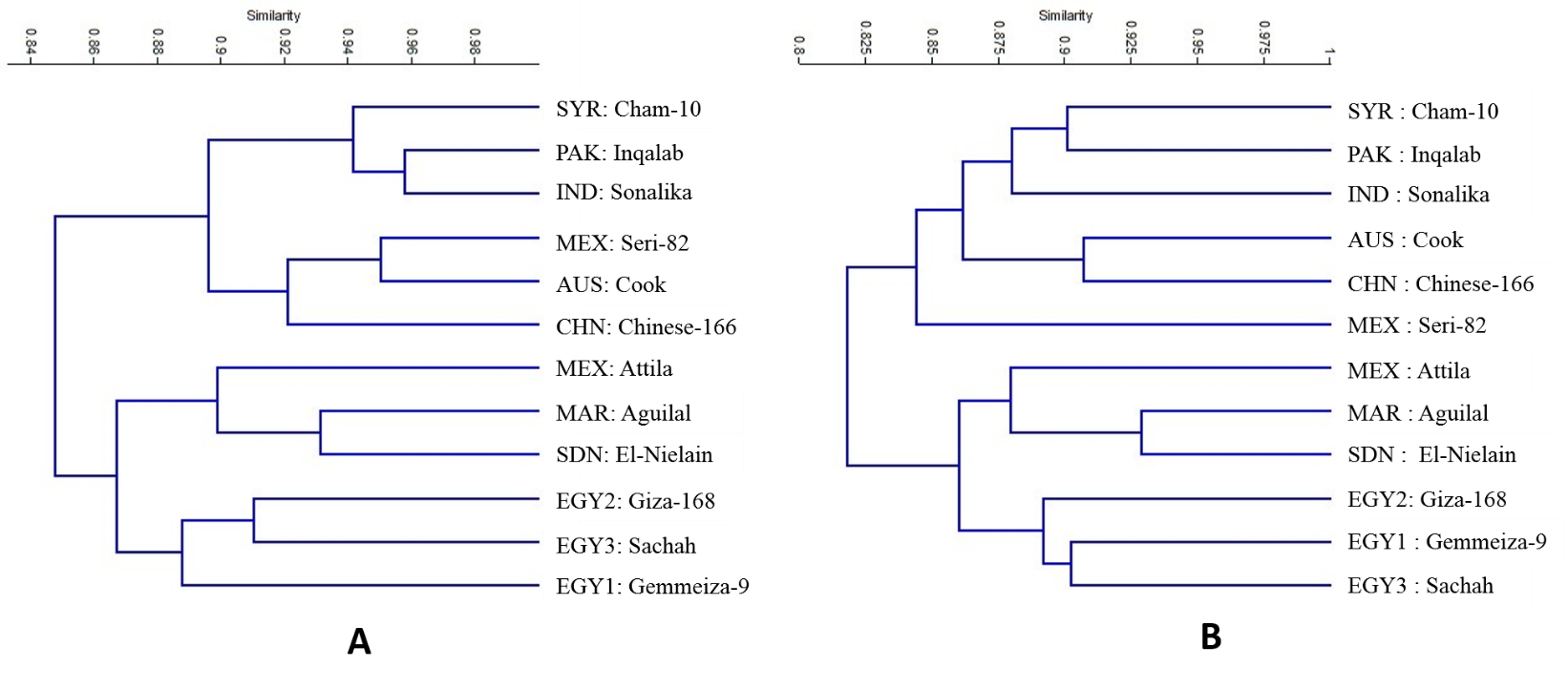


**Supplementary Fig. S3.** Genetic diversity, expressed as similarity scale, among the studied 12 wheat cultivars as revealed by (**A**) ISSR and (**B**) SCoT markers analysis using PAST Software.

**Fig. S4. (Chloroplast DNA barcoding fully uncropped figures)**


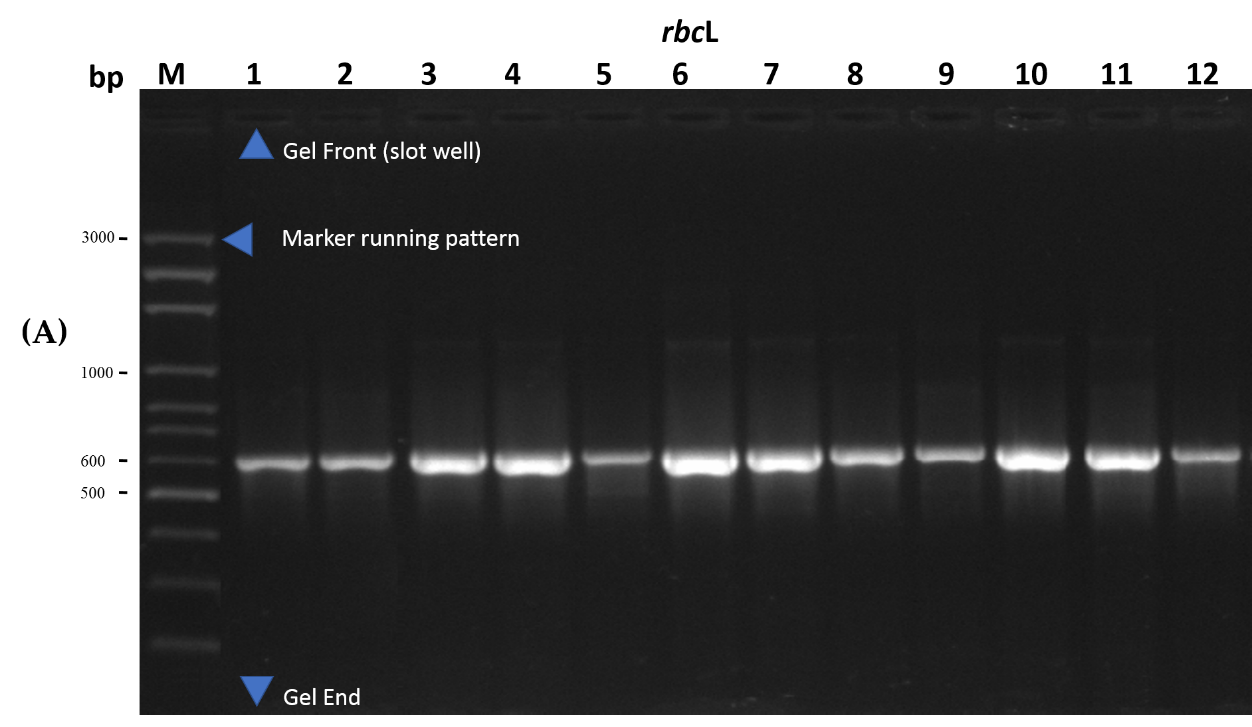

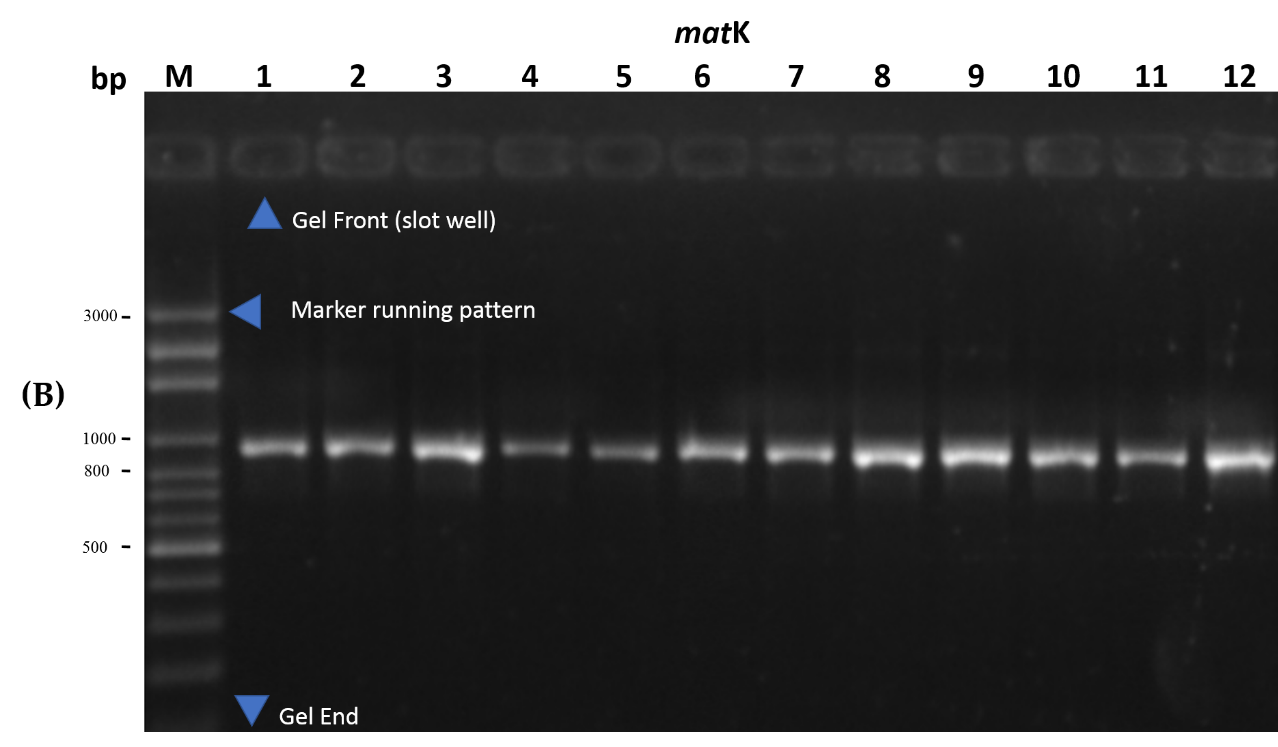


**Supplementary Fig. S4.** Amplification of DNA barcoding loci of *rbc*L and *mat*K genes. Agarose gel electrophoresis of the specific PCR products of (A) *rbc*L and (B) *mat*K was shown. Blue arrowheads indicate the gel front (starting from the slot wells), the full running pattern of the DNA size marker, and the gel end. Also, the molecular size of DNA size marker is denoted by the numbers left-handed of every agarose gel shown panel (A and B).

**Fig. S5**


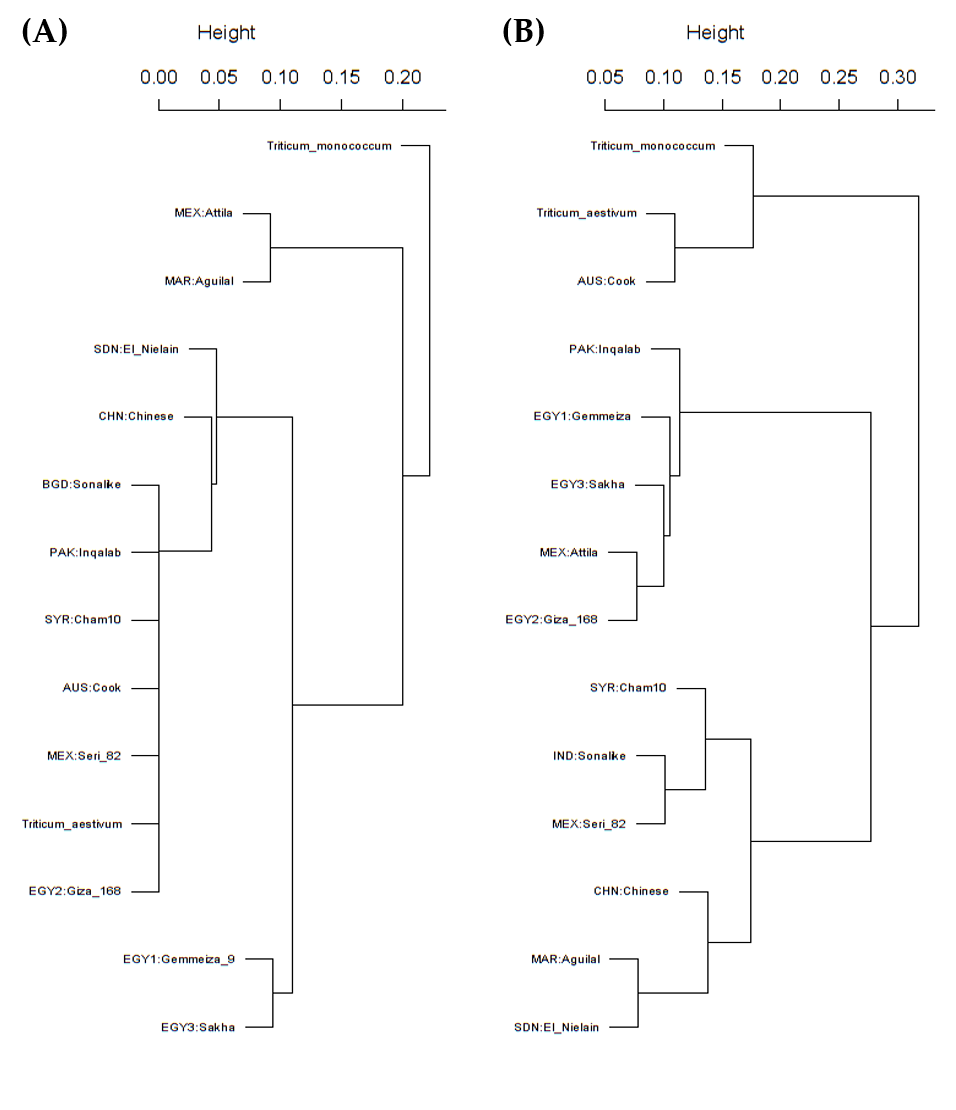
**Supplementary Fig. S5.** Cluster trees of (**A**) *rbc*L and (**B**) *mat*K genes sequence diversity illustrating the genetic diversity among the studied wheat cultivars.

**Fig. S6**


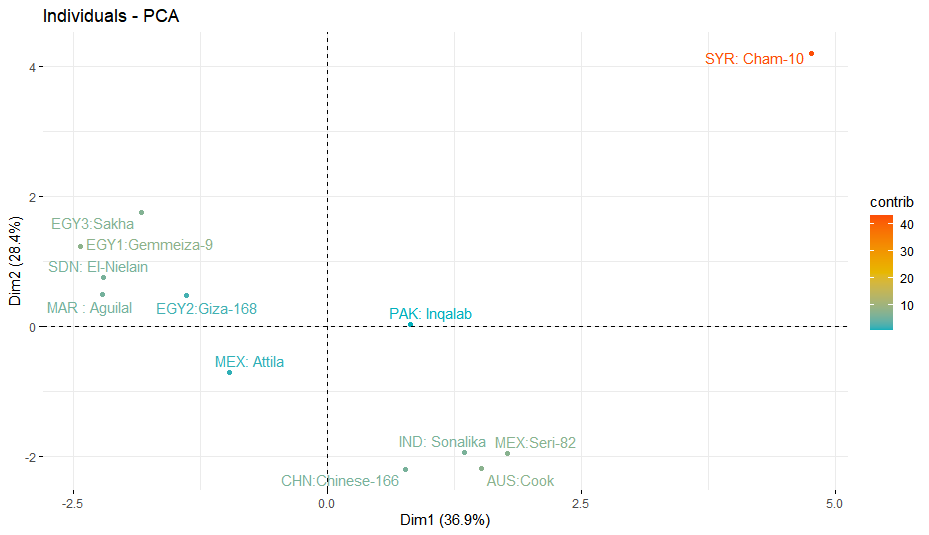


**Supplementary Fig. S6.** PCA scatter diagram showing the genetic diversity among the studied wheat cultivars as revealed by the analysis of combined ISSR and SCoT markers polymorphism and DNA sequence variation of *rbc*L and *mat*K genes using the "FactoMineR" and “Factoextra” package in R software.
